# Laboratory-based dried blood spot testing for hepatitis C: A protocol for systematic review and meta-analysis of diagnostic accuracy

**DOI:** 10.12688/hrbopenres.13166.1

**Published:** 2020-10-27

**Authors:** Paul G. Carty, Michael McCarthy, Sinead O’Neill, Patricia Harrington, Michelle O’Neill, Conor Teljeur, Susan M. Smith, Máirín Ryan

**Affiliations:** 1 Health Information and Quality Authority, Dublin, D07 E98Y, Ireland; 2 Department of General Practice, Royal College of Surgeons in Ireland, Dublin, D02 YN77, Ireland; 3 Department of Pharmacology & Therapeutics, Trinity College Dublin, Dublin, D02 PN40, Ireland

**Keywords:** Hepatitis C, HCV, dried blood spot, DBS, testing, screening, systematic review, diagnostic accuracy

## Abstract

**Background:** Diagnosis of chronic hepatitis C virus (HCV) infection typically involves collection of venous blood samples prior to serological investigation of an antibody response followed by a confirmatory viral load or antigen test to verify active HCV infection. This conventional pathway poses logistical challenges for the implementation of reflex testing, whereby the confirmatory test is performed on the same sample used for serological investigation. Dried blood spot (DBS) testing, in which capillary blood is deposited on filter paper, is a less invasive alternative that can enable reflex testing without the need for venepuncture, centrifugation and freezing of samples.

**Methods:** This systematic review aims to assess the diagnostic accuracy of DBS compared with venous blood samples for diagnosis of chronic HCV infection. Observational studies which compare diagnostic tests using DBS with those using serum, plasma or whole blood in patients with chronic or resolved HCV infection will be included. Electronic searches will be conducted in PubMed, Embase, Scopus, Web of Science, Lilacs and the Cochrane library. Citation screening, data extraction and quality appraisal of included studies will be performed in duplicate using the Quality Assessment of Diagnostic Accuracy Studies-2 (QUADAS-2) tool. A meta-analysis will be conducted to derive pooled estimates of sensitivity, specificity, positive likelihood ratios, negative likelihood ratios, and diagnostic odds ratios. Sensitivity analyses and meta-regression will also be performed. Quality of the evidence will be evaluated using the GRADE criteria.

**Discussion:** Identifying and linking people with currently undiagnosed chronic HCV infection to care is pivotal to attaining global viral hepatitis elimination targets. The use of DBS could simplify diagnostic testing strategies by integrating reflex testing into the care pathway and reducing drop-off along the cascade of care.

**Registration:** PROSPERO, CRD42020205204. Registered 19 ^th^ September 2020.

## Introduction

### Background

The hepatitis C virus (HCV), first identified in 1989, is a blood borne virus which infects the liver and commonly causes progressive liver disease ^1^. HCV is most often transmitted through injecting drug use (that is, sharing of needles and other drug paraphernalia) ^2, 3^. Although HCV infection can often resolve spontaneously, between 55–85% of people develop chronic HCV infection which may lead to liver fibrosis, cirrhosis and potentially fatal complications such as decompensated cirrhosis and hepatocellular carcinoma ^4– 8^. With recent advances in HCV therapies that are, in most cases, curative and acceptable to patients, clinical focus has shifted towards identifying and curing those currently living with undiagnosed chronic HCV infection in efforts to achieve viral hepatitis elimination targets ^2, 9^.

Diagnosis of chronic HCV infection typically involves venous blood collection prior to undergoing the following testing procedure: (1) an initial serological test to indicate an antibody response following exposure to HCV (that is, anti-HCV positive); and (2) a confirmatory viral load or antigen test to verify active HCV infection by either a nucleic acid test (NAT; to detect viral ribonucleic acid [RNA]) or a core antigen test (to detect the HCV viral protein) ^10^. Importantly, this conventional approach means that sequential healthcare attendances are generally required to provide samples and receive a diagnosis, thus increasing the risk of patient drop-off along the clinical pathway. Ultimately, such linkage-to-care issues may culminate in a missed opportunity for diagnosis and treatment ^11^. Reflex testing, whereby the confirmatory test is performed on the same sample used for the serological test, offers a potential solution to this challenge. However, if using conventional samples for reflex testing, in order to prevent sample degradation of venous serum or plasma samples, reflex testing necessitates centrifugation, freezing and cold-chain storage of samples within six to 24 hours following phlebotomy, which is often not logistically possible in community settings ^12, 13^.

Dried blood spot (DBS) testing, which involves depositing a finger prick of whole blood on filter paper, is a potential mechanism for enabling reflex testing ^11^. As DBS can be prepared using capillary blood, it circumvents the need for venepuncture, centrifugation and freezing of samples ^12^. Despite its clear logistical advantages, the diagnostic accuracy of laboratory assays using DBS for diagnosis of chronic HCV infection is subject to uncertainty. Determination of diagnostic accuracy is a key step in informing the clinical effectiveness of incorporating DBS testing into the diagnostic pathway with the aim of correctly and efficiently identifying people with currently undiagnosed chronic HCV infection. 

### Description of the intervention

For DBS testing, a skin puncture is made with a retractable lancet or a finger puncture device. Drops of blood are then applied to filter paper and dried at room temperature for up to four hours ^14^. After drying, the blood remains stable on the DBS card and can be inserted into moisture-protected packaging for transportation to a centralised laboratory. In the laboratory, the whole blood is eluted from the DBS card and the sample is run using the standard automated platforms ^11, 13, 14^. DBS facilitates the sampling process by avoiding venepuncture (that is, it is less invasive), removing the need to separate serum and plasma, requiring smaller volumes of blood and blood components, and obviating the need for cold-chain storage ^11, 15– 17^. As multiple spots can be collected at once, reflex RNA or core antigen testing can be undertaken using the second or third spot (where the initial spot is anti-HCV positive) ^18^.

### Purpose of the systematic review

Although several systematic reviews have previously been published between 2017 and 2019 ^12, 15, 19– 21^, up-to-date estimates of the diagnostic accuracy of DBS testing for detecting HCV exposure and diagnosing chronic HCV infection are warranted owing to increasing adoption and logistical applicability to clinical pathways and the publication of further studies on this topic since the original searches. Furthermore, no published systematic review has assessed the diagnostic accuracy of using DBS samples for HCV core antigen testing, to the best of our knowledge. HCV core antigen has proven to be a stable, easy to operate and affordable alternative to HCV RNA and can be regarded as an appropriate reference standard test for a HCV diagnosis ^22– 25^.

The aim of this systematic review is to assess the diagnostic accuracy of DBS samples compared with venous (whole blood, serum or plasma) blood samples for detection of HCV using laboratory-based tests (that is, anti-HCV, RNA and core antigen). The systematic review is being undertaken with a view to informing a national health technology assessment (HTA) of birth cohort testing for HCV in Ireland ^26^.

## Methods

This protocol, which follows the Preferred Reporting Items for Systematic Review and Meta-Analysis Protocols (PRISMA-P) criteria ^27^, outlines the proposed approach to systematically reviewing the available evidence on the diagnostic accuracy of DBS samples for detection of HCV using laboratory-based tests. The PRISMA-P checklist for this protocol is presented as *Extended data* ^28^.

The reporting of this systematic review will adhere to the Preferred Reporting Items for Systematic Reviews and Meta-Analysis of Diagnostic Test Accuracy studies (PRISMA-DTA) criteria ^29^. The systematic review will also conform to national HTA guidelines for evaluating the clinical effectiveness of health technologies ^30^.

### Review question

The systematic review question has been formulated using the Population, Index test, Reference test, Diagnosis (PIRD) framework and presented in Table 1 ^31^. The systematic review aims to answer:

**Table 1.** Systematic review question defined using PIRD framework.

**Population**	Adults exposed to, having or suspected of having chronic HCV
**Index test**	DBS (obtained by finger puncture and depositing blood drops on filter paper) tested for the presence of anti-HCV antibodies, HCV-RNA or HCV core antigen in a laboratory setting
**Reference test**	Venous blood (serum, plasma or whole blood) samples tested for the presence of anti-HCV antibodies, HCV-RNA or HCV core antigen in a laboratory setting
**Diagnosis of** **interest**	Diagnosis of chronic or resolved HCV infection

DBS, dried blood spot; HCV, hepatitis C virus; RNA, ribonucleic acid.

- What is the diagnostic accuracy of laboratory-based HCV testing using DBS compared with venous blood (whole blood, serum or plasma) samples among patients with chronic or resolved HCV infection?

### Eligibility criteria

Included studies must have assessed the detection of at least one of anti-HCV antibody, HCV-RNA or HCV core antigen using a DBS sample, and reported sufficient data to estimate sensitivity and specificity (that is, sufficient data must be presented to construct 2x2 tables to calculate the number of true positives (TPs), true negatives (TNs), false positives (FPs) and false negatives (FNs)). Cross-sectional and case-control studies which compare the index test (anti-HCV, HCV-RNA or HCV core antigen in DBS sample) with the reference test (anti-HCV, HCV-RNA or HCV core antigen in serum, plasma or whole blood sample) in the population of interest will be included in the systematic review.

Only data relevant to our population of interest will be extracted from studies that present data using population subgroups (for example, studies that include both adults and children). Where a receiver operating characteristic (ROC) curve analysis is used to estimate the optimal cut-off point, data will be extracted at multiple thresholds and explored in sensitivity analysis. Viral load thresholds of ≥1000 international units per milliliter (IU/mL) and ≥3000 IU/mL have been recommended when assessing the sensitivity of HCV-RNA and core antigen tests ^23, 32, 33^.

The following exclusion criteria will be applied:

- Studies in children only
- Studies that present insufficient data to construct 2×2 contingency tables to calculate the number of TPs, TNs, FPs and FNs
- Point-of-care tests conducted outside of laboratory settings that used DBS samples
- Studies where DBS results have not been compared against a reference standard method in serum, plasma or whole blood samples
- Studies in which both DBS and reference standard method have been carried out in all subjects
- Case reports, expert opinion, conference abstracts and literature reviews
- Letters to the editor and commentaries where insufficient detail on study methods and results are presented
- Animal studies
- Studies where an English translation cannot be retrieved.

### Search methods

Electronic searches will be conducted in PubMed, Embase, Scopus, Web of Science, Lilacs and the Cochrane library (which includes the Database of Systematic Reviews, the Database of Abstracts of Reviews of Effects (DARE), the Health Technology Assessment Database (HTA) and the National Health Service Economic Evaluation Database (NHS EED), supplemented by a grey literature search of national and international electronic sources. The search terms are based on those used in a previously published systematic review and are in line with Cochrane guidance for identifying diagnostic accuracy studies ^15, 34^. Forward citation searching and handsearching of the reference lists of included studies will also be conducted. The preliminary search was run from inception up to July 17 ^th^ 2020, but may be updated to include additional studies, should it be warranted. The full search strategy is presented as *Extended data* ^28^.

### Selection of studies

All citations will be screened independently by two reviewers as per the inclusion criteria, with any disagreements being resolved by discussion. Screening will be undertaken using the EndNote X8 software.

### Data extraction and management

Data extraction will be performed independently by two people with any disagreements resolved through discussion. A data extraction template, based on the standards for the reporting of diagnostic accuracy studies (STARD) checklist ^35^, is presented as *Extended data*. Key data to be extracted include:

- Population characteristics (country, sample size, age, gender, HCV genotype, fibrosis levels, treatment status, and HCV risk factors such as HCV/HIV co-infection, sexual orientation, intravenous drug misuse)
- Index test (assay type, manufacturer assay, cut-off points and limits of detection, viral load threshold, filter paper)
- Reference test (assay type, manufacturer assay, cut-off points and limits of detection)
- Outcomes (TPs, FPs, TNs, FNs)
- Setting (healthcare setting of sample collection, storage conditions and transportation, spoilage)
- Author conflicts of interest.

### Risk of bias assessment

The methodological quality of included studies will be assessed using the Quality Assessment of Diagnostic Accuracy Studies-2 (QUADAS-2) tool ^36^. Study risk of bias in each domain of QUADAS-2 will be graded low, high or some concerns. The risk of bias assessment will be conducted independently by two reviewers, with disagreements resolved through discussion.

### GRADE assessment

GRADE summary of findings tables, developed using GRADEpro software, will be presented. The body of evidence will be independently assessed by two reviewers for each primary outcome (that is, sensitivity and specificity) according to risk of bias, consistency, directness, precision and publication bias in accordance with previously published GRADE guidance ^37– 39^.

### Data synthesis and statistical analysis

All statistical analysis will be carried out in R Studio. Mean estimates and 95% confidence intervals of sensitivity, specificity, positive predictive value (PPV), negative predictive value (NPV), positive likelihood ratio (LR+), negative likelihood ratio (LR-), and diagnostic odds ratios (DORs) will be calculated for each included study. Where clinical or methodological heterogeneity precludes meta-analysis, individual mean estimates and 95% confidence intervals will be presented without pooling of effects and the evidence will be synthesised narratively. 

Data and statistical analysis will be performed according to the type of HCV detection method (that is, anti-HCV, HCV-RNA and HCV core antigen). Within each of these categories, the data may be further sub-grouped according to the type of laboratory assay (that is, enzyme-linked immunoassay (ELISA), chemiluminescence assay (CLIA), reverse-transcription polymerase chain reaction (RT-PCR), transcription-mediated amplification (TMA), etc).

If there are more than three included studies and the data are sufficiently homogenous, a meta-analysis of univariate outcomes (that is, sensitivity, specificity, PPV, NPV, LR+, LR- and DORs) will be undertaken. A random effects model will be used in the presence of statistical heterogeneity, determined using estimates of Cochran’s Q test, tau ^2^ and the inconsistency index (I ^2^). The pooled estimates will be presented on forest plots.

A random-effects bivariate model will be used to account for the correlation between pairs of sensitivity and specificity ^40^, should the included data be of sufficient quantity and quality. The outcomes of the bivariate model will be presented on summary ROC curves and compared with those of the univariate analyses. If there is evidence of a threshold effect, a hierarchical model, which allows study level covariates to be added to the model, will be considered for estimating summary ROC curves ^41^.

Subgroup analyses will be used to present sensitivity reported at alternative cut-off points, such as viral load thresholds (for example, viral load thresholds of ≥1000 IU/mL and ≥3000 IU/mL). Where corresponding values of sensitivity and specificity are reported across multiple thresholds within individual studies, meta-analysis using all available data will be reported as a sensitivity analysis ^42^. Sensitivity analysis will also be performed to detect outliers and influential studies.

Finally, heterogeneity in effect sizes will be investigated through meta-regression to estimate the effect of potential covariates (such as country-income status ^43^, the presence of co-infection, other risk HCV risk factors and prevalence) on diagnostic accuracy. Deek’s funnel plot and test for asymmetry will be used to assess publication bias ^44^. For all statistical analyses, a significant effect will be defined at a p-value of ≤0.05.

### Dissemination

The key findings of this systematic review will be published on the website of the Health Information and Quality Authority. The systematic review will also be submitted for peer-reviewed publication in international journals and submitted for presentation at national and international conferences. The data collected and/or analysed during the undertaking of this systematic review will be made available upon reasonable request to the corresponding author.

### Study status

Database searching and full-text screening against eligibility criteria are completed. Data extraction and quality appraisal are currently underway.

## Discussion

Recent advances in HCV therapies have led to a paradigm shift towards identifying and curing those currently living with chronic HCV infection with the aim of achieving the World Health Organization’s viral hepatitis elimination targets by 2030 ^2, 9^. A key logistical challenge for identifying the currently undiagnosed population is how best to integrate reflex testing into the care pathway with a view to minimising patient drop-off along the cascade of care ^11^.

As has been demonstrated by human immunodeficiency virus (HIV) testing ^12^, the use of DBS samples at central laboratories offers potential logistical advantages over the current standard of blood samples collected by phlebotomy because of enhanced stability that obviates the need for cold-chain storage, simplified transport (since current in-country transport networks can be used without separating samples for reflex testing) and ease of use for healthcare workers ^13, 14^. While no DBS assays are currently commercially available for diagnosis of HCV infection ^19, 45^, growing interest in its “off-label” use has led to the development of standardised laboratory protocols for using DBS samples in immunoassay and molecular techniques ^46^.

The aim of this systematic review is to assess the diagnostic accuracy of DBS samples compared with venous blood samples for detection of HCV using laboratory-based tests (that is, anti-HCV, RNA and core antigen). The systematic review will be undertaken with a view to informing a national HTA of birth cohort testing for HCV in Ireland ^26^.

## Data availability

### Underlying data

No underlying data are associated with this article.

### Extended data

Open Science Framework: Laboratory-based dried blood spot testing for hepatitis C: A protocol for systematic review and meta-analysis of diagnostic accuracy. https://osf.io/ghx52/files/ ^28^.

This project contains the following extended data:

- Supplementary file 1 - PRISMA-P Checklist
- Supplementary file 2 - Search strategy.pdf
- Supplementary file 3 - Data extraction template.pdf

### Reporting guidelines

PRISMA-P checklist for “Laboratory-based dried blood spot testing for hepatitis C: A protocol for systematic review and meta-analysis of diagnostic accuracy.” https://osf.io/ghx52/files/ ^28^.

Data are available under the terms of the Creative Commons Zero "No rights reserved" data waiver (CC0 1.0 Public domain dedication).
