# Supplementary material for: Laboratory-based dried blood spot testing for hepatitis C: A protocol for systematic review and meta-analysis of diagnostic accuracy
Source: HRB Open Res. 2020 Oct 27;3:78. [Version 1] doi: 10.12688/hrbopenres.13166.1 (PMC8666990; doi:10.12688/hrbopenres.13166.1)
Supplement: Peer review history

# Peer review: Reviewer response for version 1

**DOI:** 10.21956/hrbopenres.14290.r30840

**Published:** 2021-12-07

**Authors:** Manuel Hernández-Guerra

**Affiliations:** 1 Liver Unit, Hospital Universitario de Canarias, Universidad de La Laguna, Tenerife, Spain

This is a promising study that will shed light on the accuracy of DBS tests. Evidence is needed to encourage (or not) the use of this non-invasive diagnostic tool, especially where venipuncture limits the diagnosis or continuum care. The design of the study is well planned and detailed analysis is given.

**Minor issues:**

- Days from sampling to DBS processing should be registered.
- Data about quantitative correlation analysis between serum and DBS samples should be reported and if corrections were performed.

Is the study design appropriate for the research question?

Yes

Is the rationale for, and objectives of, the study clearly described?

Yes

Are sufficient details of the methods provided to allow replication by others?

Yes

Are the datasets clearly presented in a useable and accessible format?

Not applicable

Reviewer Expertise:

HCV elimination

I confirm that I have read this submission and believe that I have an appropriate level of expertise to confirm that it is of an acceptable scientific standard.

# Peer review: Reviewer response for version 1

**DOI:** 10.21956/hrbopenres.14290.r30790

**Published:** 2021-11-10

**Authors:** Nishi Prabdial-Sing

**Affiliations:** 1 Centre for Vaccines and Immunology, National Institute for Communicable Diseases, Johannesburg, South Africa

A well-written and thought out protocol for the practicalities of use of DBS instead of whole blood for HCV testing. A meta-analyses will provide insight on how accurately DBS performs when compared to reference tests on conventional sampling.

 On page 4 of 7 “Studies in which both DBS and reference standard method have been carried out in all subjects” –  why is this an exclusion? Samples will have to be tested on the reference method and index method (DBS).

 Key data to be extracted could also include: methods used to normalize serum cut-off values between reference sample (serum or plasma) and DBS for anti-HCV and HCV core antigen and IU/ml for viral load

Is the study design appropriate for the research question?

Yes

Is the rationale for, and objectives of, the study clearly described?

Yes

Are sufficient details of the methods provided to allow replication by others?

Yes

Are the datasets clearly presented in a useable and accessible format?

Not applicable

Reviewer Expertise:

Virology, viral hepatitis, diagnostics, infectious diseases, molecular biology

I confirm that I have read this submission and believe that I have an appropriate level of expertise to confirm that it is of an acceptable scientific standard.
